# The Role of *Taraxacum mongolicum* in a *Puccinellia tenuiflora* Community under Saline–Alkali Stress

**DOI:** 10.3390/molecules27248746

**Published:** 2022-12-09

**Authors:** Xueyan Lu, Yan Jin, Xiaorui Guo, Mingyuan Xu, Zhonghua Tang, Qi Chen

**Affiliations:** 1 School of Life Sciences, Nantong University, Nantong 226010, China; 2 Heilongjiang Green Food Science Research Institute, Northeast Agricultural University, Harbin 150030, China; 3 Key Laboratory of Plant Ecology, Northeast Forestry University, Harbin 150040, China; 4 First Affiliated Hospital, Heilongjiang University of Chinese Medicine, Harbin 150040, China

**Keywords:** *Taraxacum mongolicum*, saline–alkali stress, *Puccinellia tenuiflora* community, primary metabolites, elements, phenolic metabolites

## Abstract

Coexisting salt and alkaline stresses seriously threaten plant survival. Most studies have focused on halophytes; however, knowledge on how plants defend against saline–alkali stress is limited. This study investigated the role of *Taraxacum mongolicum* in a *Puccinellia tenuiflora* community under environmental saline–alkali stress to analyse the response of elements and metabolites in *T. mongolicum*, using *P. tenuiflora* as a control. The results show that the macroelements Ca and Mg are significantly accumulated in the aboveground parts (particularly in the stem) of *T. mongolicum*. Microelements B and Mo are also accumulated in *T. mongolicum*. Microelement B can adjust the transformation of sugars, and Mo contributes to the improvement in nitrogen metabolism. Furthermore, the metabolomic results demonstrate that *T. mongolicum* leads to decreased sugar accumulation and increased amounts of amino acids and organic acids to help plants resist saline–alkali stress. The resource allocation of carbon (sugar) and nitrogen (amino acids) results in the accumulation of only a few phenolic metabolites (i.e., petunidin, chlorogenic acid, and quercetin-3-O-rhamnoside) in *T. mongolicum*. These phenolic metabolites help to scavenge excess reactive oxygen species. Our study primarily helps in understanding the contribution of *T. mongolicum* in *P. tenuiflora* communities on coping with saline–alkali stress.

## 1. Introduction

Soil salinisation is a serious ecological problem that exerts complex adverse effects on plant metabolism [1]. With the aggravation of inappropriate anthropogenic activities, the natural environment has deteriorated, and soil saline-alkalisation has become increasingly serious [2]. According to statistics, approximately 7% of land worldwide (i.e., more than 900 million ha) is threatened by saline-alkalisation [3]. Plants grown on saline–alkali soil respond to both salt and alkali stress. Salt stress primarily results from NaCl, Na_2_SO_4_, and other neutral salts, producing a range of adverse effects. The excess accumulation of Cl^−^ and Na^+^ induces specific ionic toxicities and disrupts ion homeostasis [4,5,6]. High salinity decreases the osmotic potential of the soil solution, reduces the uptake of water, diminishes the photosynthetic ability of natural plants, and ultimately inhibits plant growth [4,7]. Salt stress also increases the generation of reactive oxygen species (ROS) and induces damage to intracellular components [4]. Alkali stress is primarily caused by high amounts of NaHCO_3_ and Na_2_CO_3_. High pH induces serious changes in the morphology and physiological functions of most plants [5,8]. Elevated pH levels lead to a deficiency in external protons and an increase in plant root-growth resistance, which further disturbs the ion balance in plants [8,9,10]. High pH levels also severely disturb intracellular pH stability, destroy cell membrane integrity, and impair photosynthetic function [11,12]. Soil salinisation and alkalisation are typically observed together in many cases. The coexistence of soil salinisation and alkalisation further aggravate their adverse effects [1,13].

Prior research has demonstrated that in order to adapt to salt stress, plants have developed a series of morphological, physiological, biochemical, and molecular defence mechanisms, such as maintaining ion homeostasis, regulating osmotic balance, scavenging ROS, inducing signalling transduction, modulating antioxidative enzyme activities, mediating excretion, and performing the intracellular compartmentalisation of salts [9,14,15,16,17]. Some metabolites, such as betaine, proline, polyamine, and polyhydric alcohol, are considered to be beneficial for salt-stress tolerance in plants [1]. Fructose and sucrose, signalling molecules which can stimulate related enzymes, are widely recognised as regulators of salt responses. Unsaturated free fatty acids were found to enhance the activity of plasma membrane H^+^-ATPase in *Arabidopsis* under salt stress [18,19]. Moreover, glucose was reported to improve salt tolerance by regulating the expression of the glucose sensor hexokinase1 in apple (*Malus domestica* Borkh) [20].

Plant responses to alkali stress have also been investigated. Under mild alkali stress, bermudagrass reduces carbohydrate degradation and suppresses nitrogen metabolic processes to maintain basic growth. However, under moderate and severe alkali stress, bermudagrass utilises different response strategies. Higher amounts of carbohydrates, as well as significantly elevated ROS and malondialdehyde contents, are observed [21]. After arbuscular mycorrhizal fungi inoculation, *Puccinellia tenuiflora* (*P. tenuiflora*) exhibit higher amounts of amino acids, organic acids, flavonoids, and hormones. These elevated metabolites adjust and benefit the maintenance of cell membrane stability under alkali stress [22]. The responses to salt and alkali stresses have been thoroughly investigated. In contrast, little attention has been given to responses to saline–alkali stress, although soil salinisation and alkalisation are commonly observed together [3,22].

Considering that plants collectively compose community responses to saline–alkali stress in the natural environment, it is crucial to decipher the roles of plants in their community. In this study, *Taraxacum mongolicum* (*T. mongolicum*) was selected as the experimental subject and *P. tenuiflora* was selected as a control to investigate the role of *T. mongolicum* on elements and metabolites in the *P. tenuiflora* community under saline–alkali stress. The findings may provide novel insights into saline–alkali tolerance mechanisms from the perspective of plant communities.

## 2. Results

### 2.1. Accumulation and Distribution of Elements in T. mongolicum

The rhizosphere soil around *T. mongolicum* is defined as saline–alkali soil, based on our previous research [23]. The parameters of the rhizosphere soil were as follows: pH of 8.76, exchangeable sodium percentage of 40.58%, soluble salt content of 0.57%, electrical conductivity of 895 mS/cm, (CO_3_^2−^ HCO_3_^−^)/(Cl^−^ SO_4_^2−^) ratio of 0.81, and Cl^−^/SO_4_^2−^ ratio of 0.37.

**Figure 1:**
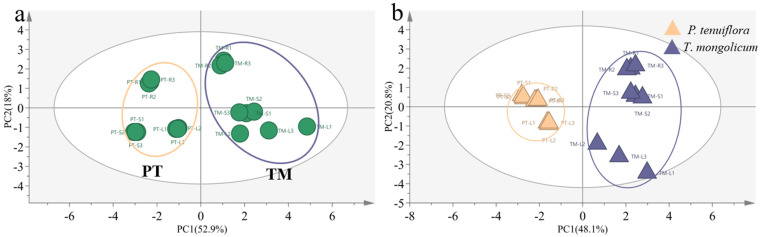
Comparison of elements between *T*. *mongolicum* and *P*. *tenuiflora*. (**a**) PCA score plot. Purple circle: *T*. *mongolicum*, orange circle: *P*. *tenuiflora*. TM: *T*. *mongolicum* group, PT: *P*. *tenuiflora* group. (**b**) OPLS−DA score plot. Purple triangle: *P*. *tenuiflora*, orange triangle: *T*. *mongolicum*.

**Figure 2:**
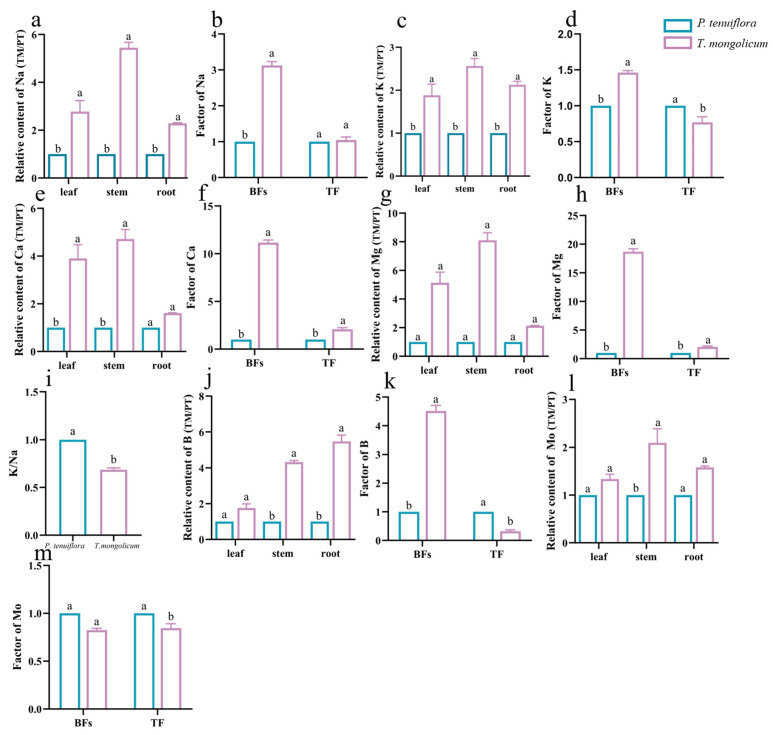
Relative abundances of significantly different elements in *T*. *mongolicum*. (**a**,**c**,**e**,**g**,**j**,**l**) Relative contents of (**a**) Na; (**c**) K; (**e**) Ca; (**g**) Mg; (**j**) B; and (**l**) Mo in *T*. *mongolicum* compared to their contents in *P*. *tenuiflora*. The contents of elements in *P*. *tenuiflora* are normalized to 1. (**b**,**d**,**f**,**h**,**k**,**m**) Factors of (**b**) Na; (**d**) K; (**f**) Ca; (**h**) Mg; (**k**) B; and (**m**) Mo in *T*. *mongolicum* compared to factors in *P*. *tenuiflora*. The factors of elements in *P*. *tenuiflora* are normalized to 1. (**i**) The ratio of Na and K. Element contents and factors are summarized from 3 biological replicates and presented as mean ± SEM. Different letters indicate significant differences between groups (*p*-value < 0.05).

The results from the PCA plot and the OPLS-DA plot identified the diverse expression patterns of elements in *P. tenuiflora* and *T. mongolicum* (VIP > 1, *p*-value < 0.05) (Figure 1). The elements K, Ca, Na, Mg, B, and Mo were differentially expressed between *P. tenuiflora* and *T. mongolicum*, and significantly accumulated in *T. mongolicum* (Figure 2). The measurements of elements in different tissues (leaf, stem, and root) showed that Na contents in the leaf, stem, and root of *T. mongolicum* increased 2.8-fold, 5.4-fold, and 2.3-fold, respectively (Figure 2a), compared with *P. tenuiflora*. Calculated factors demonstrated that Na was absorbed from soil by *T. mongolicum* (Figure 2b). In addition to Na, macroelements K, Ca, and Mg were evidently accumulated in the aboveground parts of *T. mongolicum*, particularly in the stem (Figure 2c–h). This viewpoint was further supported by the ratio of K/Na in *T. mongolicum* being evidently higher than that in *P. tenuiflora* (Figure 2i). Ca and Mg play important roles in resisting the toxicity of Na in *T. mongolicum*. The relative contents of Ca and Mg were even larger than that of K in *T. mongolicum*. In *T. mongolicum*, Ca contents increased 3.9-fold and 4.7-fold in the leaf and stem, respectively (Figure 2f). Mg contents increased 5.1-fold and 8.1-fold in the leaf and stem, respectively, in *T. mongolicum* compared with those in *P. tenuiflora* (Figure 2g). These significantly accumulated macroelements in *T. mongolicum* were primarily actively absorbed from the soil. The microelements B and Mo were also significantly accumulated in *T. mongolicum* (Figure 2j–m). The calculated relative contents of B indicated that B was primarily distributed in the stem and root. BF and TF values revealed that B was absorbed from the soil and accumulated in the root. Mo was transported from the root to the leaf (Figure 2k,m).

### 2.2. Responses of Primary Metabolites to Saline–Alkali Stress

**Figure 3:**
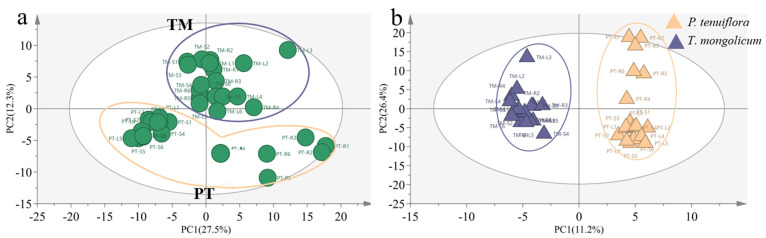
Comparison of primary metabolites between *T*. *mongolicum* and *P*. *tenuiflora*. (**a**) PCA score plot. Purple circle: *T*. *mongolicum*, orange circle: *P*. *tenuiflora*. TM: *T*. *mongolicum* group, PT: *P*. *tenuiflora* group. (**b**) OPLS−DA score plot. Purple triangle: *P*. *tenuiflora*, orange triangle: *T*. *mongolicum*.

**Figure 4:**
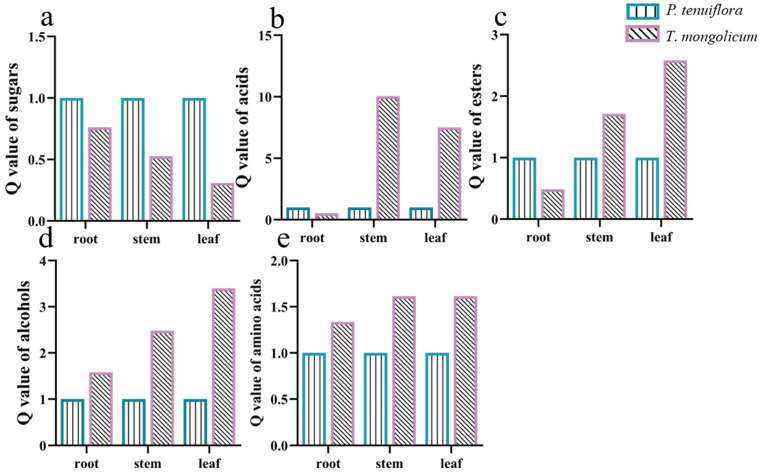
Q-values of significantly different metabolites in *T*. *mongolicum*. Relative Q-values of (**a**) sugars; (**b**) acids; (**c**) esters; (**d**) alcohols; and (**e**) amino acids in *T*. *mongolicum* compared to Q-values in *P*. *tenuiflora*. Q-values of metabolites in *P*. *tenuiflora* are normalised to 1.

In total, 226 effective compounds were detected in the leaf, stem, and root of *P. tenuiflora* and *T. mongolicum* via GC-MS. Similar to the PCA score plot of elements in *P. tenuiflora* and *T. mongolicum*, the PCA score plot of primary metabolites exhibited robust differences between *P. tenuiflora* and *T. mongolicum* (Figure 3a). The OPLS-DA score plot showed a PC1 of 11.2% and a PC2 of 26.4%, revealing a clear distinction between *P. tenuiflora* and *T. mongolicum* (Figure 3b). Among these 226 compounds, 88 primary metabolites were significantly differentially expressed between *P. tenuiflora* and *T. mongolicum*, screened by a threshold of VIP > 1 and *p*-value < 0.05. These significantly different metabolites could be divided into 13 sugars, 10 alcohols, 7 amino acids, 13 esters, 28 acids, and 17 other compounds (not shown) (Table 1). Calculated Q-values indicated that sugars were significantly accumulated in *P. tenuiflora* (Figure 4a); acids and esters were significantly accumulated in the aboveground parts of *T. mongolicum* (Figure 4b,c); and alcohols and amino acids were markedly accumulated in all parts of *T. mongolicum* (Figure 4d,e). These findings indicated that there might be variable distributions of energy and materials between *P. tenuiflora* and *T. mongolicum*.

**Figure 5:**
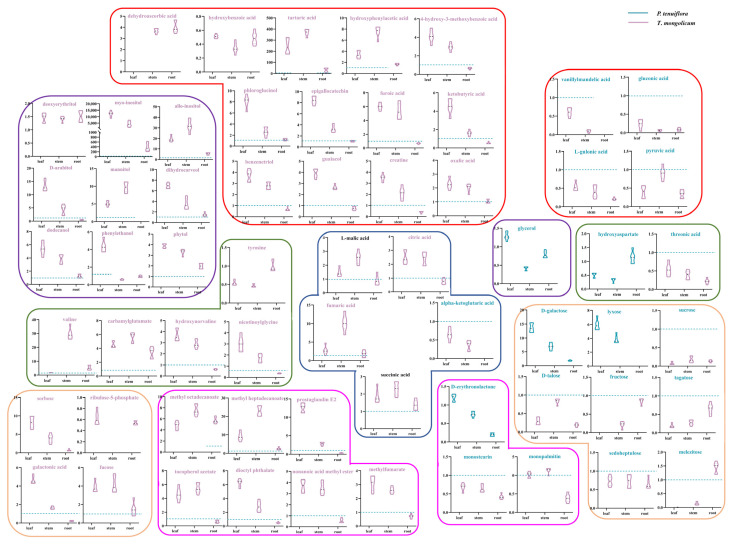
The metabolic networks of significantly different primary metabolites. The contents of primary metabolites in *P*. *tenuiflora* are normalised to 1 and represented as dashed lines in blue. Purple frame represents alcohols, orange frame represents sugars, green frame represents amino acids, rose pink frame represents esters, red frame represents acids, and dark blue frame represents components in the TCA cycle. Pink title indicates that the contents of primary metabolites in *T*. *mongolicum* are higher than in *P*. *tenuiflora* while blue title represents that the contents of primary metabolites in *P*. *tenuiflora* are higher than in *T*. *mongolicum*. Black title indicates that the primary metabolite was not a significantly different metabolite.

To explore the metabolic mechanisms underlying variable distributions of energy and materials, metabolic pathways of different tissues under saline–alkali stress were visualised (Figure 5). Amino acid metabolism was more activated in *T. mongolicum*. Valine exhibited a 1.7-fold increase in the leaf, a 29.8-fold increase in the stem, and a 5.2-fold increase in the root of *T. mongolicum*, compared with those of *P. tenuiflora.* Nicotinoylglycine and hydroxynorvaline markedly accumulated in the leaf of *T. mongolicum*, exhibiting a 3.0-fold increase and a 3.7-fold increase, respectively. Nicotinoylglycine and hydroxynorvaline also exhibited a 1.7-fold increase and a 2.9-fold increase in the stem of *T. mongolicum*, respectively. Carbamylglutamate exhibited a 4.5-fold increase in the leaf, 5.6-fold increase in the stem, and 5.2-fold increase in the root of *T. mongolicum*, compared with those of *P. tenuiflora.* Tyrosine only accumulated in *T. mongolicum*, exhibiting a relative content of 0.5 in the leaf, 0.5 in the stem, and 1.0 in the root (Figure 5).

Unlike amino acid metabolism, sugar metabolism was more involved in *P. tenuiflora*, indicating a choice between carbon metabolism (represented by sugar) and nitrogen metabolism (represented by amino acid) in plants via the tricarboxylic acid (TCA) cycle in response to saline–alkali stress. Among them, fumaric acid and citric acid were significantly different metabolites. Fumaric acid increased by 2.7-fold (leaf), 10.1-fold (stem), and 1.8-fold (root) in *T. mongolicum*, whereas citric acid increased by 2.5-fold (leaf) and 2.4-fold (stem) in *T. mongolicum*. For sugar metabolism, in *T. mongolicum*, galactonic acid exhibited a 4.6-fold increase in the leaf and a 1.7-fold increase in the stem, whereas fucose exhibited a 3.9-fold increase in the leaf and a 4.1-fold increase in the stem. Sorbose and ribulose-5-phosphate were only accumulated in *T. mongolicum* (Figure 5).

The metabolites of alcohols, esters, and acids are involved in carbon–TCA–amino acid metabolism as supplements. More alcohols were enriched in *T. mongolicum*, increasing 12,102-fold (leaf), 4314-fold (stem), and 388-fold (root) for myo-inositol; 19.4-fold (leaf), 32.1-fold (stem), and 5.5-fold (root) for allo-inositol; 13.3-fold (leaf) and 4.4-fold (stem) or D-arabitol; 5.1-fold (leaf) and 9.7-fold (stem) for mannitol; 7.0-fold (leaf) and 3.5-fold (stem) for dihydrocarveol; 5.4-fold (leaf) and 3.9-fold (stem) for dodecanol; 4.3-fold (leaf) for phenylethanol; and 3.9-fold (leaf) and 3.3-fold (stem) for phytol, compared with those in *P. tenuiflora*. Moreover, deoxyerythritol was only enriched in *T. mongolicum*. Esters were more accumulated in the aboveground parts of *T. mongolicum*. Specifically, methyl heptadecanoate exhibited 9.1-fold (leaf), 23.0-fold (stem), and 2.4-fold (root) increases in *T. mongolicum*; prostaglandin E2 exhibited 12.7-fold (leaf) and 2.8-fold (stem) increases; tocopherol acetate exhibited 4.6-fold (leaf) and 5.4-fold (stem) increases; dioctyl phthalate exhibited 6.3-fold (leaf) and 2.9-fold (stem) increases; nonanoic acid methyl ester exhibited 3.7-fold (leaf) and 3.5-fold (stem) increases; and methylfumarate exhibited 3.1-fold (leaf) and 2.6-fold (stem) increases. Methyl octadecenoate only accumulated in the aboveground parts of *T. mongolicum* (Figure 5).

Acids involved in sugar–TCA–amino acid metabolism were also massively accumulated in the aboveground parts of *T. mongolicum*, increasing 243.6-fold (leaf), 367.5-fold (stem), and 30.4-fold (root) for tartaric acid; 3.4-fold (leaf) and 7.5-fold (stem) for hydroxyphenylacetic acid; 4.1-fold (leaf) and 3.0-fold (stem) for 4-hydroxy-3-methoxybenzoic acid; 8.1-fold (leaf) and 2.6-fold (stem) for phloroglucinol; 8.4-fold (leaf) and 3.3-fold (stem) for epigallocatechin; 6.0-fold (leaf) and 5.3-fold (stem) for furoic acid; 4.5-fold (leaf) and 1.6-fold (stem) for ketobutyric acid; 3.7-fold (leaf) and 2.8-fold (stem) for benzenetriol; 3.9-fold (leaf) and 2.6-fold (stem) for guaiacol; 3.5-fold (leaf) and 2.1-fold (stem) for creatine; and 2.3-fold (leaf) and 2.0-fold (stem) for oxalic acid, compared with those in *P. tenuiflora*. Hydroxybenzoic acid and dehydroascorbic acid were only accumulated in *T. mongolicum*.

### 2.3. Responses of Phenolic Metabolites to Saline–Alkali Stress

**Figure 6:**
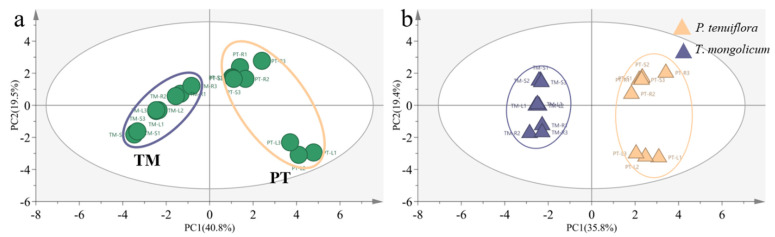
Comparison of phenolic metabolites between *T*. *mongolicum* and *P*. *tenuiflora*. (**a**) PCA score plot. Purple circle: *T*. *mongolicum*, orange circle: *P*. *tenuiflora*. TM: *T*. *mongolicum* group, PT: *P*. *tenuiflora* group. (**b**) OPLS−DA score plot. Purple triangle: *P*. *tenuiflora*, orange triangle: *T*. *mongolicum*.

**Figure 7:**
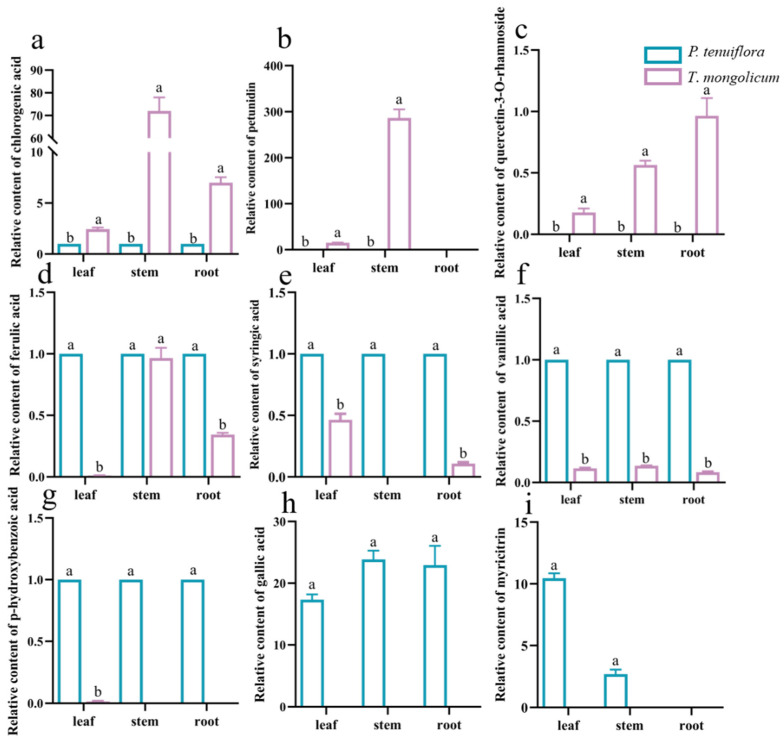
Relative contents of significantly different phenolic metabolites in *T*. *mongolicum*. Relative contents of (**a**) chlorogenic acid; (**b**) petunidin; (**c**) quercetin-3-O-rhamnoside; (**d**) ferulic acid; (**e**) syringic acid; (**f**) vanillic acid; (**g**) p-hydroxybenzoic acid; (**h**) gallic acid; and (**i**) myricitrin in *T*. *mongolicum* compared to their contents in *P*. *tenuiflora*. The contents of phenolic metabolites in *P*. *tenuiflora* are normalised to 1. Phenolic metabolite contents are summarised from 3 biological replicates and presented as mean ± SEM. Different letters indicate significant differences between groups (*p-*value < 0.05).

To further explore the response mechanisms of *T. mongolicum* against saline–alkali stress, the relative abundances of phenolic metabolites were summarised. In total, 20 effective phenolic metabolites were detected from 34 phenolic metabolites. Among these 20 effective phenolic metabolites, 9 significantly different phenolic metabolites were screened, based on the PCA plot and OPLS-DA score plot between *P. tenuiflora* and *T. mongolicum* (Figure 6, Table 2). These phenolic metabolites were accumulated less in *T. mongolicum* than in *P. tenuiflora*. Among these phenolic metabolites, chlorogenic acid was significantly accumulated in *T. mongolicum*, increasing 7.0-fold in the leaf, 72.0-fold in the stem, and 2.4-fold in the root compared with *P. tenuiflora* (Figure 7a). In the aboveground parts of *T. mongolicum*, petunidin increased 15.0-fold in the leaf and 286.6-fold in the stem compared with *P. tenuiflora* (Figure 7b). Another prominent compound, quercetin-3-O-rhamnoside, was only detected in *T. mongolicum* (Figure 7c). The other PCs were more markedly accumulated in *P. tenuiflora* than *T. mongolicum* (Figure 7d–i).

## 3. Materials and Methods

### 3.1. Plant Materials and Growth Conditions

*T. mongolicum* and *P. tenuiflora* were gathered from saline–alkaline land in the Hulun Buir Grassland, Inner Mongolia Autonomous Region, China. Chen Qi identified *T. mongolicum* and *P. tenuiflora*. *T. mongolicum* belongs to the *P. tenuiflora* community, which is one of the dominant community types in the Hulun Buir Grassland. These two plants were collected from three different sample plots and stored at low temperature (<4 °C) for further study. *P. tenuiflora* was considered as the control group.

#### Measurement of Elemental Contents

Tissue samples (leaf, stem, and root) were dried at 60 °C and digested in concentrated HNO_3_ (95%) using a graphite plate (EH45A plus, Shanghai, China) at 130 °C. The elemental contents (Na, K, Ca, Mg, B, Fe, Mn, Ni, Mo, Cu, and Zn) were determined using an inductively-coupled plasma-emission spectrometer (ICP-OES Optima 8000, Perkin Elmer, Boston, MA, USA) and calculated from the standard curve of each element. The factors of elements, including the bioaccumulation factor (BF) and transfer factor (TF), were calculated according to a previously described method [24], where:

BF = Mplant/Mmedium;
TF = Mleaf/Mroot; and

Mplant: mass of element in plant (mg); Mmedium: mass of element in medium (mg); Mleaf: mass of element in leave (mg); Mroot: mass of element in root (mg).

### 3.2. Measurement of Primary Metabolites

The extraction and determination of primary metabolites were performed as described by Chen et al. with a few improvements [24]. Briefly, 90 mg of the sample was weighed, and 600 μL extract solution (including 540 μL cold methanol and 60 μL internal standard) was added. After 30 min of ultra-sonication, 300 μL chloroform and 600 μL water were added to the mixture. After 2 min of vortexing, the mixture was ultra-sonicated for 30 min and centrifuged at 13,000× *g* rpm at 4 °C for 10 min. Afterwards, 700 μL supernatant was transferred to a glass vial and dried in a vacuum concentrator. Dried samples were reconstituted by adding 400 μL methoxyamine (15 mg/mL in pyridine). The obtained solution was derived through sequential reaction with 400 μL BSTFA (15 mg/mL in 1% trimethylcholorosilane) and 80 μL n-hexane for GC-MS analysis.

Extracted samples were injected into the GC-MS system (Agilent 7890A-5975C, Agilent Technologies, Inc., Santa Clara, CA, USA). The constant flow rate was 1.0 mL/min, and the model of the non-polar DB-5 capillary column was 30 m × 250 μm I.D. (J&W Scientific, Folsom, CA, USA). The scanning range was set from 50 to 500 *m*/*z*, and the electron impact (EI) ion source was maintained at 70 Ev. The quality control sample was prepared by mixing the aliquots of tissues samples together to produce a pooled sample.

### 3.3. Measurement of Phenolic Metabolites

The extraction and determination of phenolic metabolites were performed as described by Chen et al. [24]. Briefly, 1.0 g of the sample was homogenised with 10 mL 70% methanol, ultra-sonicated for 40 min, and centrifuged at 8000× *g* rpm for 10 min. The supernatant was retained, the residual residue was re-extracted, and the supernatant was mixed and dried under vacuum twice. The dried sample was re-dissolved in 1 mL 70% methanol and filtered with 0.22 μm nylon membrane for ultra-high-performance liquid chromatography quadrupole time-of-flight mass spectrometry (UPLC-qTOF-MS) analysis. The UPLC (Waters, Tokyo, Japan) conditions were as follows: A%: 0.05% formic acid-water, B%: 0.05% formic acid–acetonitrile, and ACQUIT UPLC-BEH C18 Column (1.7 mm, 2.1 mm × 50 mm, Waters, Milford, MA, USA). The volume injected was 2 μL, and the column temperature was maintained at 30 °C. Mass spectrometry (Waters^®^ Xevo G2 QTOF mass spectrometer, Waters) conditions were as follows: positive ion mode, capillary voltage of 3.0 kV, cone voltage of 45 V, source temperature of 400 °C, and desolvation temperature of 500 °C. The scanning range was set between 50 and −1000 *m*/*z*, with an ion-acquisition rate of 0.2/s. Extracted samples were injected into the mass spectrometer every 10 s at a flow rate of 5 μL/min. Leu-enkephalin was applied as an internal standard.

### 3.4. Statistical Analysis

The GC-MS data were analysed using Chroma TOF software (LECO, San Joes, CA, USA). The UPLC-qTOF-MS data were analysed and normalised using MassLynxTM (Waters). Unsupervised principal component analysis (PCA) and orthogonal partial least squares discriminant analysis (OPLS-DA) were performed to visualise the differences between groups. Significantly different compounds were screened by Student’s t-test and the multivariate statistical method (*p*-value < 0.05 and VIP > 1.0). The metabolic pathway was analysed by Kyoto Enrichment of Genes and Genomes (KEGG). The data underwent log2 transformation and min–max normalisation for improving data normality. Data were presented as the mean ± standard error (SEM). The score of principal component “Q” was calculated using SPSS (IBM, New York, NY, USA). Boxplot and histograms were generated using GraphPad Prism8 (Harvey, GraphPad Software, San Diego, CA, USA). Pathway maps were drawn with Adobe illustrator (Adobe, San Diego, CA, USA).

## 4. Discussion

Soil alkalisation is a major abiotic stress that affects plant growth, vegetation distribution, and crop yield [19,25]. Saline–alkali stress damages the physiological condition of plants, interrupts biochemical metabolism, and may even lead to the plant’s death [3,26,27]. Plant communities are naturally distributed in the saline–alkali soil and normally function as a team to resist saline–alkali stress. However, the roles of plants in the community under saline–alkali stress are typically neglected. In this study, the accumulation of elements, carbon–TCA–nitrogen metabolites, and phenolic metabolites were analysed to explore the role of *T. mongolicum* in a *P. tenuiflora* community under saline–alkali stress.

Compared with *P. tenuiflora*, Na was significantly accumulated in the aboveground parts of *T. mongolicum*, indicating that saline–alkali stress disrupts the ionic balance and induces Na toxicity. Maintaining high K and low Na concentrations is the main mechanism of plants to maintain osmotic regulation and ensure the proper functioning of many enzymes [1]. Several studies have demonstrated that competitive relationships exist between K and Na during their uptake under the conditions of high salt and alkali stress. The amount of Na increased, whereas the total K content decreased [1]. In this study, K was significantly accumulated in *T. mongolicum*, indicating a potential lack of competitive inhibition between the absorption of Na and K, which may be due to the recretohalophyte attributes of *T. mongolicum*. These kinds of plants may have a unique pathway of Na absorption, independent of K. Our findings suggest that in *T. mongolicum*, K cannot fight Na toxicity independently.

The macroelements Ca and Mg were enriched in the aboveground parts of *T. mongolicum*, indicating that they play a key role in resisting Na toxicity. Ca is a key signalling component of the salt-overly sensitive (SOS) pathway, which contributes to the maintenance of K/Na balance by extruding Na [28]. Our results showed that Ca is massively absorbed from soil and is accumulated in the aboveground parts of *T. mongolicum*, particularly in the stem. The distribution of Ca was consistent with that of Na, indicating that saline–alkali stress may activate the SOS–Na system to exclude Na and diminish the damage caused by Na toxicity.

Superfluous Na can destroy the structures and suppress the functions of chloroplasts, negatively affecting the photosynthetic capacity and chlorophyll content [1,29]. Mg is a central element of Mg-protoporphyrin and is thus involved in the biosynthesis of chlorophyll. In this study, Mg was significantly accumulated in the aboveground parts of *T. mongolicum*, indicating that the photosynthesis of *T. mongolicum* is not affected by saline–alkali stress. Mg was absorbed from the soil and transported to the aboveground parts of *T. mongolicum* to regulate chlorophyll biosynthesis and the photosynthetic rate. This suggests that *T. mongolicum* has relatively strong Mg-uptake capability. Mg content was considered as a signal arising from pools of adenylates, could reflecting lower energy status (less ATP) of a tissue in response to salt stress [30,31]. In our studies, leaves and stem have a lower-energy status. Saline–alkali stress may activate Mg-uptake channels, reduce the accumulation of Na, and improve osmotic adjustment. Remarkably, Na, Ca, and Mg were significantly accumulated in the stem of *T. mongolicum*, compared with *P. tenuiflora*, implying that the stem is the defensive battlefield of *T. mongolicum* in response to Na toxicity.

Microelement B can promote sugar transformation and transportation. Additionally, it forms peroxides to improve oxygen supply to plant roots [32]. It also plays an important role in regulating water distribution in plants [32,33]. In this study, *T. mongolicum* accumulated more B than *P. tenuiflora* under saline–alkali stress, implying that sugar metabolites and water distribution are regulated to respond to saline–alkali stress in *T. mongolicum*. In addition, B can limit vegetative growth by regulating the contents of phenolic metabolites under stress. Compared with *P. tenuiflora*, in *T. mongolicum*, B accumulated in the root. Accumulated B may improve the supply of oxygen and H_2_O_2_ and regulate phenolic metabolites in response to stress in the leaf. The microelement Mo is an important component of nitrogenase and nitrate reductase that affects plant nitrogen metabolism [34]. Our results show that a higher amount of Mo was accumulated in *T. mongolicum* than in *P. tenuiflora*, indicating that nitrogen metabolism plays an important role in the response of *T. mongolicum* to saline–alkali stress.

Sugar metabolism and nitrogen metabolism were highlighted in this study using the microelements B and Mo. To investigate the relationship between sugar metabolism and nitrogen metabolism under saline–alkali stress, primary metabolites were detected via GC-MS. The metabolic networks of some significantly different primary metabolites, including sugars, amino acids, esters, and acids, were also established. In this study, amino acids and esters accumulated more in the aboveground parts of *T. mongolicum*, whereas sugars accumulated more in *P. tenuiflora*. This demonstrates that a different response strategy is present. Under saline–alkali stress, plants can alter the contents of metabolites associated with sugar metabolism and change amino acid synthesis as well as the TCA cycle to counteract stress [35]. The concentrations of sugars, such as glucose, fructose, and sucrose, were increased in response to neutral salt stress [35]. These sugars are enhanced under neutral salt stress, implying that the degradation of polysaccharides as a carbon source likely promotes the maintenance of osmotic balance. Nevertheless, the concentrations of sugars were significantly reduced in response to saline–alkali stress; this was caused by the inhibition of reducing forces and the limitation of nitrogen metabolism [1,36]. This is similar to our findings in the *P. tenuiflora* community. Our results suggest that *P. tenuiflora* improves saline–alkali stress via the selection of metabolic strategies for other plants within the community. We speculate that nitrogen metabolism and ester metabolism are used by *T. mongolicum* to further respond to saline–alkali stress.

Previous studies have indicated that amino acids serve as key precursors for the synthesis of some secondary metabolites involved in plant defence responses [37]. At high pH levels, alkaline salt stress leads to the reduced production of amino acids and limited nitrogen intake/transport and metabolism [1,38,39,40]. In this study, amino acids were significantly accumulated in *T. mongolicum*, indicating that nitrogen metabolism still plays an important role in the defence against saline–alkali stress. The accumulation of amino acids can improve plant tolerance to saline–alkali stress by mediating the removal of ROS [7,41]. The accumulation of amino acids is a critical mechanism to compensate cellular osmolarity [42]. A choice exists between carbon and nitrogen metabolism, and *T. mongolicum* is more inclined to undergo nitrogen metabolism compared with *P. tenuiflora*.

Fatty acids are also significantly accumulated in the aboveground parts of *T. mongolicum*. Fatty acids reserve energy to participate in sugar–TCA–nitrogen metabolism. Fatty acids and their derivatives are also involved in plant resistance to abiotic stress [43,44]. As important components of the cytomembrane, polyunsaturated fatty acids play an important role in maintaining and regulating the normal biological functions of cells [17,45].

The accumulation of organic acids may be important for plants to adapt to saline–alkali stress [5,46]. In this study, acids were significantly accumulated in the aboveground parts of *T. mongolicum*. These organic acids play a key role in regulating pH. The accumulation of organic acids can reduce cell water potential and maintain the balance of ions and pH in cells [22,40,43,47]. In *P. tenuiflora*, organic acids are primarily accumulated in the root. Increased organic acid secretion may increase the acidity of the rhizosphere, thereby neutralising alkalinity surrounding the plant roots and promoting root growth [48]. In addition, alcohols, considered as osmotic regulators, were significantly accumulated in *T. mongolicum*. These alcohols can maintain osmotic pressure balance and scavenge ROS. Phenolic metabolites are essential secondary metabolites. In plants, they play a vital role against abiotic stress [49,50,51]. Phenolic metabolites and their derivatives reportedly improve soybean salt tolerance [52]. Similar results have also been reported in other species. In *Arabidopsis*, increasing the levels of flavonoids by transgenes can increase the tolerance to salt stress [53,54,55]. To investigate the role of phenolic metabolites in *T. mongolicum* under saline–alkali stress, the levels of phenolic metabolites were detected. The condition of surplus carbon in plants is a beneficial signal for phenolic metabolism. Therefore, the accumulation of phenolic metabolites is increased more when plants have higher carbohydrate reserves [56,57]. Consistent with the observed accumulation of sugars in *P. tenuiflora*, we found that phenolic metabolites accumulated more in *P. tenuiflora*. This observation further reflects the distribution trade-off between carbon and nitrogen in plants under saline–alkali stress [58,59]. Sugar accumulation also provides more carbon resources for specialised metabolites in plants. From a total of 20 phenolic metabolites, only petunidin, chlorogenic acid, and quercetin-3-O-rhamnoside were significantly accumulated in *T. mongolicum*. These phenolic metabolites were highlighted in conditions of limited resource distribution, demonstrating that they are crucial components in dealing with saline–alkali stress. Petunidin, quercetin-3-O-rhamnoside, and chlorogenic acid are established phenylpropanoid compounds which play a positive role in scavenging excess ROS. Therefore, accumulated petunidin, quercetin-3-O-rhamnoside, and chlorogenic acid may benefit *T. mongolicum* in the regulation of osmotic balance [15,52]. Phenolic metabolites can enhance plant tolerance to abiotic and biotic stresses as they can remove dangerous stress-response substances, including free radicals, singlet oxygen molecules, and peroxides, from the cell [22,60]. Some phenolic metabolites are found to have a strong binding affinity to superoxide dismutase 1 (SOD1). These phenolic metabolites stabilise the SOD1 dimer and inhibit the aggregation of SOD1 [60,61]. Phenolic metabolites and their derivatives play critical roles as antioxidants in response to saline–alkali stress [55,62]. Therefore, in *T. mongolicum*, petunidin, chlorogenic acid, and quercetin-3-O-rhamnoside may play an important role in ameliorating saline–alkali stress [63].

## 5. Conclusions

*T. mongolicum* plays an important role in the *P*. *tenuiflora* community in balancing the elements Ca, Mg, B, and Mo; the metabolism of nitrogen and fatty acids; as well as the accumulation of organic acids, metabolic solutes, and some phenolic metabolites. The macroelements Ca and Mg are accumulated in the aboveground parts, particularly in the stem of *T. mongolicum*, to defend against Na toxicity. B accumulated in *T. mongolicum* regulates sugar transformation and transportation, whereas Mo affects nitrogen metabolism. Compared with *P. tenuiflora*, *T. mongolicum* decreases the accumulation of carbohydrates, rather than synthesising more amino acids and organic acids, to help resist saline–alkali stressed environments. Enriched petunidin, chlorogenic acid, and quercetin-3-O-rhamnoside in *T. mongolicum* play positive roles in scavenging excess ROS.

## Figures and Tables

**Table 1:** List of significantly different primary metabolites between *T*. *mongolicum* and *P*. *tenuiflora*.

	Metabolites	VIP	*p*-Value
sugars	tagatose	1.70	***
	sorbose	1.67	**
	sucrose	1.60	***
	melezitose	1.56	***
	D-talose	1.46	***
	fucose	1.30	**
	D-galactose	1.26	**
	fructose	1.19	***
	lyxose	1.14	**
	ribulose-5-phosphate	1.13	**
	sedoheptulose	1.08	**
	galactonic acid	1.05	*
	lactobionic acid	1.00	**
amino acids	threonic acid	1.44	**
	tyrosine	1.40	***
	carbamylglutamate	1.33	***
	hydroxyaspartate	1.27	**
	valine	1.13	**
	hydroxynorvaline	1.05	**
	nicotinoylglycine	1.02	**
alcohols	allo-inositol	2.08	****
	myo-inositol	2.06	****
	phytol	1.74	***
	phenylethanol	1.70	****
	dodecanol	1.42	***
	dihydrocarveol	1.27	**
	glycerol	1.31	***
	deoxyerythritol	1.18	**
	D-arabitol	1.08	*
	mannitol	1.04	**
esters	tocopherol acetate	1.38	**
	monostearin	1.36	***
	monopalmitin	1.28	***
	D-erythronolactone	1.25	**
	methyl heptadecanoate	1.21	**
	methylfumarate	1.17	**
	dioctyl phthalate	1.05	**
	methyl octadecanoate	1.05	**
	nonanoic acid methyl ester	1.04	**
	stearic acid	1.02	**
	prostaglandin A2	1.01	**
	palmitic acid	1.01	**
	prostaglandin E2	1.00	**
acids	hydroxyphenylacetic acid	1.89	****
	5-hydroxyindole-2-carboxylic acid	1.64	***
	tartaric acid	1.43	****
	alpha-ketoglutaric acid	1.36	***
	benzenetriol	1.34	***
	gluconic acid	1.32	**
	hydroxybenzoic acid	1.30	***
	phloroglucinol	1.24	***
	mucic acid	1.23	***
	trans-muconic acid	1.23	***
	fumaric acid	1.23	**
	pyruvic acid	1.22	***
	oxalic acid	1.21	***
	guaiacol	1.20	***
	glycerol 1-phosphate	1.18	**
	vanillylmandelic acid	1.17	**
	dehydroascorbic acid	1.17	**
	ketobutyric acid	1.16	**
	L-gulonic acid	1.14	**
	4-hydroxy-3-methoxybenzoic acid	1.13	**
	hydroxybenzoic acid	1.11	**
	epigallocatechin	1.10	**
	creatine	1.08	**
	nicotinic acid	1.07	**
	lactic acid	1.06	**
	citraconic acid	1.03	**
	furoic acid	1.01	**
	hydrocinnamic acid	1.01	**

VIP, variable importance in the projection; * *p*-value < 0.05; ** *p*-value < 0.01; *** *p*-value < 0.001; **** *p*-value < 0.0001. Data are summarized from 6 biological replicates.

**Table 2:** List of significantly different phenolic metabolites between *T*. *mongolicum* and *P*. *tenuiflora*.

Phenolic Metabolites	VIP	*p*-Value
vanillic acid	1.40	****
*p*-hydroxybenzoic acid	1.30	****
gallic acid	1.23	****
chlorogenic acid	1.18	****
syringic acid	1.17	****
myricitrin	1.09	**
petunidin	1.09	**
quercetin-3-O-rhamnoside	1.06	**
ferulic acid	1.02	*

VIP: variable importance in the projection; * *p*-value < 0.05; ** *p*-value < 0.01; **** *p*-value < 0.0001. Data are summarised from 3 biological replicates.

## Data Availability

Data related to this study is available from the corresponding author upon reasonable request.

## Sample Availability

Samples of the compounds significantly different TIAs or PCs are available from the authors.
